# The ionic DTI model (iDTI) of dynamic diffusion tensor imaging (dDTI)

**DOI:** 10.1016/j.mex.2014.09.004

**Published:** 2014-09-26

**Authors:** Nikos Makris, Gregory P. Gasic, Leoncio Garrido

**Affiliations:** a Harvard Medical School, Department of Psychiatry, Center for Morphometric Analysis, HST Athinoula A. Martinos Center, Massachusetts General Hospital, Boston, MA 02129, USA; b Harvard Medical School, Department of Neurology, Center for Morphometric Analysis, HST Athinoula A. Martinos Center, Massachusetts General Hospital, Boston, MA 02129, USA; c Harvard Medical School, Department of Radiology, HST Athinoula A. Martinos Center, Massachusetts General Hospital, Boston, MA 02129, USA; d Department of Physical Chemistry, Instituto de Ciencia y Tecnología de Polímeros, Consejo Superior de Investigaciones Científicas (ICTP-CSIC), Juan de la Cierva 3, E-28006 Madrid, Spain

**Keywords:** The ionic DTI model (iDTI) of dynamic diffusion tensor imaging (dDTI), Diffusion, Tractography, Magnetic resonance imaging, Dynamic diffusion tensor MRI, Ionic diffusion tensor MRI, Functional DTI, Functional diffusion imaging, Ionic DTI model

## Abstract

Measurements of water molecule diffusion along fiber tracts in CNS by diffusion tensor imaging (DTI) provides a static map of neural connections between brain centers, but does not capture the electrical activity along axons for these fiber tracts. Here, a modification of the DTI method is presented to enable the mapping of active fibers. It is termed dynamic diffusion tensor imaging (dDTI) and is based on a hypothesized “anisotropy reduction due to axonal excitation” (“AREX”). The potential changes in water mobility accompanying the movement of ions during the propagation of action potentials along axonal tracts are taken into account. Specifically, the proposed model, termed “ionic DTI model”, was formulated as follows.

• First, based on theoretical calculations, we calculated the molecular water flow accompanying the ionic flow perpendicular to the principal axis of fiber tracts produced by electrical conduction along excited myelinated and non-myelinated axons.
• Based on the changes in molecular water flow we estimated the signal changes as well as the changes in fractional anisotropy of axonal tracts while performing a functional task.
• The variation of fractional anisotropy in axonal tracts could allow mapping the active fiber tracts during a functional task.

Although technological advances are necessary to enable the robust and routine measurement of this electrical activity-dependent movement of water molecules perpendicular to axons, the proposed model of dDTI defines the vectorial parameters that will need to be measured to bring this much needed technique to fruition.

## Method details

### Rationale of DTI modification: the ionic DTI model

Herein, we put forward the hypothesis that task-dependent neural stimulation modifies the macromolecular and ionic environments of axonal water molecules, resulting in increased water movement across the membrane through open ion channels (Na^+^, K^+^), and we formulate a model elucidating this mechanism. As this postulated increase in water mobility would be anisotropic and prevail in the plane perpendicular to the major axis of the axon, we further hypothesize that a diffusion tensor imaging (DTI) signal decrease should be observed with neuronal activation such as during the performance of a functional task. We named this hypothesis “anisotropy reduction due to axonal excitation” or AREX. Schematically, the quantitative model that reflects AREX and represents these processes within a test region of interest (ROI) would be as follows: TASK → ACTION POTENTIALS → WATER FLOW THROUGH OPEN ION CHANNELS → INCREASE OF WATER MOBILITY BETWEEN INTRA- and EXTRA-AXONAL COMPARTMENTS → CHANGES IN *D*_app_, *λ*_2_, *λ*_2_, *λ*_3_ and *FA* (i.e., *D*_app_ increases; *λ*_1_ decreases or remains relatively unchanged; *λ*_2_ and *λ*_3_ increase; *FA* decreases) (see Fig. 1).

We called this model of dynamic DTI (dDTI), “ionic DTI model” or “iDTI”, given that water exchange through open ion channels is the physiological basis for the hypothesized diffusion anisotropy changes during axonal excitation. Unlike functional MRI (fMRI), which indirectly models the connections between disparate cortical/subcortical centers, dDTI aims to measure changes in water mobility associated water movement through open cation channels, thereby providing direct measurements of electrical activity in these connections. Thus, we will be able to characterize the precise path of axonal conduction between the centers during neuronal activation as in a dynamic task.

The biological and biophysical model iDTI is formulated in the theoretical context of a motor task in humans. According to the model proposed herein, a reduction in *FA* should be observed during the performance of this task due to the increased mobility of water flowing through open ion channels perpendicular to the direction of the axons conducting the action potentials. This trans-axonal membrane exchange of water takes place at the membrane surface of unmyelinated axons and at discrete sites along the main fiber axis of myelinated axons, i.e., at the nodes of Ranvier. At the same time, glial cells in the extra-axonal space would contribute to maintain the ions/water equilibrium needed for axon conduction. Overall, the water movement would be incoherent at macroscopic level (i.e., at the dimensions of a typical voxel size in an MRI experiment) and would be indistinguishable from “true” diffusion. However, only the exchange between intra- and extra-axonal compartments would contribute to the reduction of *FA* since, to a first approximation, the possible contribution of glial cells could be considered isotropic. Based on these and other theoretical assumptions, we modeled the physiological mechanisms underlying axonal and fiber tract excitation, and the diffusion tensor imaging processes required to detect a DTI signal. Due to the ubiquity of water, the model can be applied in both, myelinated fibers at the nodes of Ranvier and unmyelinated fibers. Also, it applies to the glial cells (oligodendrocytes and astrocytes) present in the extra-axonal space. Herein, we present this physiological model as the conceptual basis for dynamic DTI, whose implementation will require technological advances in MRI by individuals seeking to obtain non-invasive direct measurements of electrical activity between cortical/subcortical centers with the goal of determining differences between active and inactive as well as normal and disease states.

### Computation of the ionic DTI model

The model of ion and water displacement during neuronal excitation in myelinated and unmyelinated axons is grounded on basic anatomical, physiological and physical–chemical principles, logical assumptions and accepted values for parameters derived from the literature. Then, we computed the fraction of water through the ion channels (with the greatest streaming potentials) during axonal excitation for the period equivalent to that used for the collection of diffusion imaging data.

During an action potential, the largest flux of ions is due to that of sodium. Sodium fluxes in model sodium channels are accompanied by the streaming of two to three water molecules [1]. Thus, in order to assess the vectorial exchange of water along an excited fiber tract for the duration of a neuroimaging experimental observation, we modeled this exchange for saltatory (which occurs in myelinated axons at the nodes of Ranvier), and non-saltatory (which concerns unmyelinated axons) neural conduction. Glial cells are considered as buffer of the electric charge/mass in the extra-axonal space to maintain the necessary conditions for neural conduction, however the exchange is assumed to be isotropic and does not contribute to the overall changes in anisotropy. This mixed model was used to compute the total water exchange across the Na^+^ and K^+^ channels due to neural conduction in a fiber tract, considering separately each contribution, as follows.

In myelinated axons at the nodes of Ranvier, the model takes into account: (1) the number of myelinated axons in a given excited fiber bundle; (2) the number of nodes of Ranvier per mm of axon; (3) the surface area of a node of Ranvier, and (4) the density of sodium channels at these nodes.

Similarly, in unmyelinated axons, the model takes into account: (1) the number of unmyelinated axons in a given excited fiber bundle; (2) the surface area of an unmyelinated axon, and (3) the density of sodium channels at these surface areas.

In addition, we assumed for both myelinated and unmyelinated axons that: (1) the maximum flux of sodium ions per unit time has an equivalent accompaniment of water molecules as that measured during conditions of osmotic stress; (2) the overwhelming inward-flow of sodium, which is accompanied by 2–3 water molecules per sodium ion [2,3] is vectorially canceled-out in terms of electrical charge by the flow of potassium ions [4–8] that is accompanied by 0.9 to 1.4 water molecules per ion [9], in the opposite direction; (3) the total inward-flow of water molecules is compensated by an equal amount of outward-flow of water, and (4) transient changes in charge and mass occurring between the intra- and extra-axonal spaces and glial cells, re-equilibrate relatively fast compared with the time scale of the MR experiment. It could be a short-lived and topographically discrete swelling of the axon at the node of Ranvier (in the myelinated axons) or re-polarized regions along its axis (in the unmyelinated axons) and of glial cells occurring within the observation time. Nevertheless, it should be taken into consideration that to enable and maintain repetitive axonal conduction of action potentials, the ionic concentration in the axon environment must be tightly controlled [10].

Thus, the ion-related molecular water flow ($F_{w}^{MA}$) in a sample volume in myelinated axons (MA), which is defined as the isotropic volume having a cross-section equal to that of the axonal bundle of interest, was calculated according to the equation:

(1) $$F_{w}^{MA}=2N_{e}^{MA}N_{NR}S_{NR}\rho_{NR}^{cNa^{+}}N_{w}^{Na^{+}}/t$$

where $N_{e}^{MA}$ represents the number of excited myelinated axons in the bundle, *N*_*NR*_ is the number of nodes of Ranvier in these axons per sample volume, *S*_*NR*_ is the surface area of a node of Ranvier, $\rho_{NR}^{cNa^{+}}$ is the density of sodium ion channels on the membrane in a node of Ranvier, $N_{w}^{Na^{+}}$ is the number of water molecules associated to a sodium ion and *t* represents time. The factor of 2 derives from the assumption that the number of water molecules accompanying the inward ionic flow (related to Na^+^ primarily) is equal to the number of water molecules accompanying the outward ionic flow (related to K^+^ primarily). Given a balance of matter equal to zero, i.e., the inward-flow of water equals the outward-flow of water, the summation of these two opposite flows is twice the size of the flow in each direction. Eq. (1) provides the flow of water expressed as number of molecules per unit time, which multiplied by the time the system is being observed in a diffusion imaging experiment, i.e., the diffusion time gives the amount of water molecules associated with an activation event within the defined sample volume.

Similarly, the computation of molecular water flow per sample volume $F_{w}^{nMA}$ across the axonal membrane during neural conduction in unmyelinated axons (nMA) is as follows

(2) $$F_{w}^{nMA}=2N_{e}^{nMA}S_{nMA}\rho_{nMA}^{cNa^{+}}N_{w}^{Na^{+}}/t$$

where $N_{e}^{nMA}$ represents the number of excited unmyelinated axons in the bundle, *S*_*nMA*_ is the membrane area of an unmyelinated axon in the sample volume, $\rho_{nMA}^{cNa^{+}}$ is the density of sodium ion channels on the membrane in unmyelinated axons, and $N_{w}^{Na^{+}}$ and *t*, as indicated earlier.

The computation of total molecular water flow (*F*_*w*_) per sample volume within a fiber tract is derived by the summation of the water flow as expressed in Eq. (1) for the myelinated axons and in Eq. (2) for the unmyelinated axons as follows in Eq. (3):

(3) $$F_{w}=F_{w}^{MA}+F_{w}^{nMA}=2(N_{e}^{MA}N_{NR}S_{NR}\rho_{NR}^{cNa^{+}}+N_{e}^{nMA}S_{nMA}\rho_{nMA}^{cNa^{+}})N_{w}^{Na^{+}}/t$$

### Determination of FA variation

We modeled the water displacements associated with the ion flow during axonal excitation for the hand representation in the corticospinal tract in a diffusion MRI experiment. Our estimates were based on published data as shown in detail in Table 1. Assuming there are about 10^6^ axons present in the corticospinal tract (CST) with a mean diameter of 3 μm [11,12], the dimensions of an isotropic sample volume rendering a cross-section to contain the whole tract would be 3.5 mm × 3.5 mm × 3.5 mm, where a distribution of axon diameters [13] is considered to be packed as a regular array of cylinders in a square lattice with an interaxonal space of 17%. Assuming 70% of the CST axons are myelinated (∼700,000) and 30% are unmyelinated (∼300,000) [12–14], two distinct computations should be done, one for each type of axonal fibers. Thus, for myelinated axons Eq. (1) will be used. Assuming an internodal distance proportional to the axon diameter, ∅_*a*_, ∼100 × ∅_*a*_ [15], the average number of nodes of Ranvier per sample volume would be equal to 1.28 × 10^7^. To estimate the number of sodium channels per node of Ranvier, a node surface area is calculated for each axon diameter (assuming a length of node equal to 2 μm) [16], and an average density of these channels equal to 10,000 per μm^2^ [16]. Therefore, the total number of node of Ranvier sodium channels per sample volume would be 1.61 × 10^12^. If the number of water molecules that accompany a sodium ion is 2.5 and the ionic flow is 8.8 × 10^3^ sodium ions per millisecond [16], the resulting in-flow in the sample volume is about 3.53 × 10^16^ water molecules per ms or 1.06 × 10^−6^ g of water per ms.

For unmyelinated axons Eq. (2) will be used. To estimate the number of sodium channels per unmyelinated axon, a mean membrane area of 33,000 μm^2^ per axon is considered (mean axon diameter equal to 3 μm [17] and length of axon equal to 3.5 mm – the voxel length), and an average density of these channels equal to 200 per μm^2^ [17]. Thus, the total number of sodium channels per sample volume would be 1.98 × 10^12^. If, as indicated above, the number of water molecules that accompany a sodium ion is 2.5 and the ionic flow is 8.8 × 10^3^ sodium ions per millisecond [16], the resulting in-flow in the sample volume is about 4.35 × 10^16^ water molecules per ms or 1.30 × 10^−6^ g of water per ms.

Therefore, the total in-flow of water molecules per ms in the sample volume could be determined by adding the contributions of the myelinated axons and unmyelinated axons, Eq. (3), and it would be equal to 7.89 × 10^16^ water molecules or 2.36 × 10^−6^ g of water per ms. This represents the amount of water associated to the Na^+^ inward-flow, but an equal amount of water should leave the axon (outward-flow) associated to the K^+^ out-flow to prevent excessive axon swelling. Consequently, the total flow of water molecules per ms *F*_*w*_ is 2 × 7.89 × 10^16^ or 2 × 2.36 × 10^−6^ g of water per ms.

In order to get an estimate of the change that could be expected in the **D** tensor due to neuronal activation, several assumptions were made. As the proposed measurements of the **D** tensor are performed with no a priori orientation specified, none of these assumptions compromises the generality of the model. First, we assumed that the main fiber axis is aligned with the *z*-axis of the laboratory reference frame defined by the orthogonal set of gradient coils *XYZ*, and the imaging and crusher gradients have negligible effect. Hence, the diffusion coefficients along the *xyz* coordinates would correspond to the eigenvalues of the **D** tensor, *λ*_1_, *λ*_2_ and *λ*_3_, with *λ*_1_ > *λ*_2_ ≥ *λ*_3_. Second, as a first approximation we hypothesized that *λ*_1_ ≡ *D*_||_ remains practically unchanged during inactive and active states, $D_{\left|\right|}=D_{\left|\right|}^{*}$ (active state denoted by an asterisk). The rationale for this assumption is based on the fact that the impulse conduction in nerve fibers occurs by an increase in ionic and water transport across the axonal membranes, at re-polarized regions in unmyelinated axons and at the nodes of Ranvier in myelinated ones. Third, we assumed that the diffusion coefficients in the plane normal to the main fiber axis, *λ*_2_ and *λ*_3_ ≡ *D*_⊥_, are equal and the magnitude of the change between active and inactive states, $D_{⊥}^{*}-D_{⊥}$, is the same in *λ*_2_ and *λ*_3_. Then, the macroscopic average diffusion coefficient (apparent diffusion coefficient) in inactive state could be expressed as follows:

(4) $$D_{\text{app}}=(1/3)(D_{\left|\right|}+2D_{⊥})$$

and in active state as

(5) $$D_{\text{app}}^{*}=(1/3)(D_{\left|\right|}^{*}+2D_{⊥}^{*})\approx (1/3)(D_{\left|\right|}+2D_{⊥}^{*})$$

Due to their low permeability, the diffusion of water in white matter is restricted primarily by the axonal membranes [18]. Based on the model proposed by Szafer et al. [19] to describe the diffusion of water in tissues, Ford and Hackney [13] showed in a spinal cord white matter model that at long diffusion times, *Δ* ≥ 20 ms, the main factors contributing to the apparent diffusion coefficient are the permeability of the membranes and the inter-axonal space. Assuming the inter-/intra-axonal space fractions do not change significantly during axonal activation and taking into account that the diffusion time in the measurements performed here exceeds the 20 ms indicated above, we hypothesize that because of the large change in axonal membrane permeability to Na^+^ during nerve impulse conduction [20], the change in *D*_app_ from inactive to active state would be mainly due to the fraction of fast moving (activated) water, $f_{\text{w}}^{*}$, associated with the ion flux occurring between inter- and intra-axonal spaces in a voxel. This could be expressed as follows:

(6) $$D_{⊥}^{*}=f_{\text{w}}^{*}{D^{\prime }}_{⊥}+(1-f_{w}^{*})D_{⊥}$$

where ${D^{\prime }}_{⊥}$ represents the diffusion coefficient associated to the fraction of fast moving water. Assuming, as stated earlier, that $D_{\left|\right|}^{*}=D_{\left|\right|}$ and considering $f_{\text{w}}^{*}\ll 1$, from Eq. (5) results:

(7) $$D_{\text{app}}^{*}=(1/3)[D_{\left|\right|}+2(f_{\text{w}}^{*}{D^{\prime }}_{⊥}+D_{⊥})]$$

This model could be expanded taking into consideration few characteristics of tissue microstructure, as described in a recent review by Nilsson et al. [21], provided the hardware available for clinical diffusion MRI allows probing the influence of specific tissue features. Also, as indicated later, the blood–oxygen-level-dependent (BOLD) effect on these measurements must be taken into account or a strategy designed to minimize them.

For the simplified model described here, if *D*_||_ = 1 × 10^−9^ m^2^ s^−1^ and *D*_||_/*D*_⊥_ = 5 [13], the magnitude of the change in *D*_app_ caused by nerve impulse transmission could be derived by estimating $f_{\text{w}}^{*}$ and ${D^{\prime }}_{⊥}$. To calculate $f_{\text{w}}^{*}$ in the experiment described here, we take into account *F*_w_ and that the firing rate of the motor neuron, 15 Hz on average [22–24], leading to a mean duration of an action potential of 67 ms and that sampled every TR of 2.5 s. Since, as calculated above, *F*_w_ is 4.71 × 10^−6^ g ms^−1^ in the sample volume, the amount of fast moving water for a diffusion time of 35 ms would be 35 × 4.71 × 10^−6^ g or 1.65 × 10^−4^ g. Assuming white matter composition about 90% water [25] and a tissue density of ∼1 g cm^−3^, the weight of water in the 42.87 mm^3^ sample volume would be 0.039 g and the $f_{\text{w}}^{*}$ would be 4.28 × 10^−3^.

To estimate the diffusion coefficient of fast moving water, ${D^{\prime }}_{⊥}$, we would consider that during depolarization/re-polarization the inter-/intra-axonal compartments are in fast exchange at the nodal (myelinated axons) and membrane activated (unmyelinated axons) regions, behaving as if no membrane was present. Thus, to a first approximation, the diffusion coefficient of activated water would be that of free water at 37 °C, 3 × 10^−9^ m^2^ s^−1^ [26]. Then, from Eq. (6), $D_{⊥}^{*}$ would be equal to 2.13 × 10^−10^ m^2^ s^−1^ and, therefore, the change in *D*_⊥_ from inactive to active state would be 6.4%. Since we assumed no change in *D*_||_ during axonal impulse conduction, from Eq. (5), $D_{\text{app}}^{*}=4.75\times 10^{-10}{\text{ m}}^{2}{\text{ s}}^{-1}$. This represents an increase in the observed average diffusion coefficient in the active with respect to the inactive state of 1.8%.

Based on the experimental conditions outlined herein and to a first approximation, the white matter in inactive state behaves as a two compartment model in slow exchange; in other words, the diffusion coefficients in the parallel and perpendicular directions to the main fiber axis are the result of contributions from the intra- and inter-axonal compartments. Thus, to get an estimate on the effect of the predicted change in *D*_⊥_ upon the observed signal intensity, the following expression could be used provided that *D*_⊥_ represents the average (weighted inter- and intra-axonal contributions) diffusion coefficient

(8) $$A(g_{⊥})=A(0)\;\text{exp}[-(bD_{⊥})]$$

where *A*(*g*_⊥_) and *A*(0) are the amplitude of the echo in the presence of a gradient pulse with amplitude *g* perpendicular to the main fiber axis and 0, respectively, *b* = (*γgδ*)^2^ (*Δ* − *δ*/3) where *γ* is the gyromagnetic ratio of the nucleus being observed, *δ* represents the duration of the gradient pulse and *Δ* is the time between the rising edges of the gradient pulses. A change in *D*_⊥_ from 2.00 × 10^−10^ to 2.13 × 10^−10^ m^2^ s^−1^ would cause a drop in the amplitude of the echo, *A*(*g*_⊥_), of about 0.77% for *b* values of about 600 s mm^−2^. This would be just above the detection limit for imaging data with a SNR of 400. These limiting conditions for the detection of an MR signal change associated with a nerve impulse during the performance of a given task were reached by considering a total density of Na^+^ channels involved, $\rho^{Na^{+}}$, of 3.58 × 10^12^ for a sample volume of 42.87 mm^3^ or 8.35 × 10^10^ Na^+^ channels/mm^3^.

Also, it is possible to estimate the change in the fractional anisotropy, *FA*, using the following expression and the corresponding values estimated above for the inactive, *FA*, and active, FA*, states:

(9) $$FA={\left(3/2\right)}^{0.5}{\left[({\left(D_{\left|\right|}-D_{\text{app}}\right)}^{2}+2{\left(D_{⊥}-D_{\text{app}}\right)}^{2})/(D_{\left|\right|}^{2}+2D_{⊥}^{2})\right]}^{0.5}$$

(10) $$FA^{*}={\left(3/2\right)}^{0.5}{\left[({\left(D_{\left|\right|}^{*}-D_{\text{app}}^{*}\right)}^{2}+2{\left(D_{⊥}^{*}-D_{\text{app}}^{*}\right)}^{2})/(D_{\left|\right|}^{*2}+2D_{⊥}^{*2})\right]}^{0.5}$$

Thus, *FA* = 0.770 and FA* = 0.754, which represents a 2.1% reduction in the value of *FA* due to nerve impulse conduction.

## Additional information

Blood–oxygen-level-dependent (BOLD) fMRI has allowed the characterization of the neural activity in the brain systems performing motor, sensory, cognitive, and affective operations [27–30]. However, the propagation of neural activity between cortical areas along axonal pathways is not captured by fMRI measurements. Their understanding could potentially provide important insights into the integration of neural activity into systems. Likewise, it would enable the diagnosis and monitoring of potential therapeutic interventions aimed at restoring brain functions due to changes in axonal conduction. In this context, the analysis of fMRI data using network or graph theory is receiving considerable attention [31–33]. These studies aim at determining the connectivity between active nodes in the brain while performing a functional task, but they may not be mapping the actual anatomic connectivity.

MRI has shown that the anisotropy of water movement in tissues is associated with the presence of arrays of intra- and extra-cellular structures (i.e., mainly in white matter axonal membranes and myelin sheets) restricting and hindering the random movement of water [18]. These observations have been exploited to develop MR methods that map the distribution of white matter fiber tracks in the central nervous system (CNS). These are known as diffusion tensor MRI (DTI) [34–39], or MR tractography [40–42]. Whereas DTI provides anatomical information about the fiber tracks and CNS, functional deficits can only be inferred from DTI data. The existing technique does not measure electrical conduction deficits in these nerve fibers. As described above, the generation of action potentials in activated neurons involves significant ion and water transport across axonal membranes. MRI measurements of these movements have the potential to provide information about nerve conduction along these axons.

Over the past two decades there has been debate and controversy as to whether a diffusion imaging method will permit the direct imaging of action potentials in bundles associated with the performance of a functional task. Unfortunately, the published evidence does not provide a biophysical mechanism for the observed changes in DTI signal associated with the performance of a task, nor is the evidence incontrovertible.

Small changes in the apparent diffusion coefficient of water, *D*_app_, associated to activation of neurons in the visual cortex have already been observed [43]. This increase in the value of *D*_app_ of water has been attributed to the swelling of neuronal bodies during membrane repolarization by ion transport between the extra- and the intra-cellular spaces [43] or to a vascular contribution during hypercapnia [44].

Early imaging investigations failed to detect any change in the anisotropic diffusion of water in nerves using an in vitro preparation. Using an excised frog spinal cord and sciatic nerve, Gulani et al. [45] found no significant difference of the diffusion coefficient in the directions perpendicular or parallel to the direction of the nerve between the stimulated and the inactive states. They also suggested that positive results might be a misinterpretation of an underlying vascular BOLD effect as blood flow and volume increase. On the other hand, their outcome could be due to several factors. First, the level of ATP required to sustain in vivo-like electrical activity would seem to preclude a reproducible measurement over the duration of the experiments – although the authors provided some electrophysiological data no direct correlations between electrophysiological measurements and diffusion measurements were shown in the paper. Second, the diffusion time used (i.e., 11.5 ms) was very short as compared to what is used in clinical scanners (i.e., 50–70 ms). Third, the temperature of 13 degrees Celsius used in their experiment is very low as compared to 37 degrees Celsius of routine in vivo conditions. The last two factors reduce considerably the sensitivity of anisotropy and the observed difference in ADC (by a factor of 4 or 5). Nevertheless, although this experiment published negative results, it was an important step to address this question in some detail. Future studies aimed at demonstrating that a drop of the signal in diffusion anisotropy is due to a diffusion effect need to assure that this drop is decoupled from a possible BOLD effect [46].

Another paper by Anderson et al. [47] used isolated optic nerves to claim there were no large changes in anisotropic diffusion as a function of depolarization. We believe the data in that paper do not adequately address the predictions of this model in vivo. First, Anderson et al. used isolated optic nerves that have the same limitations noted with regards to ATP levels and temperature as discussed above. In the Anderson et al. paper the authors did not perform any electrophysiological experiments to demonstrate that action potentials could be sustained or generated with their preparations. Instead they used perturbations that are well-known to affect the osmolarity of the glial cells. Therefore, the negative results they obtained using a “depolarizing solution” to change *FA* were never verified as to actually producing a depolarization. The other perturbations they performed were to examine the effects of osmotic changes upon *D*_||_ and *D*_⊥_, which they, not surprisingly, found had a large effect on *D* values. The effects they observed were largely confined to the glial cells. These artificial preparations are actually quite a ways off from what one might observe in vivo, indeed, the magnitude of the osmotic perturbations used in the paper would almost certainly not be found in vivo except in conditions like stroke or massive ATP depletion. Thus, it remains to be determined whether or not proof for changes in *FA* may occur with stimulation in vivo.

To determine whether MR signal measures may or may not reflect electrical conduction along nerve tracts, a physiological model, which takes into account ion and water movements accompanying action potentials, would be critical in setting forth the parameters and goals would have to be met to insure that the observed MR signal changes during a functional task come from electrical conduction along nerve tracts. Mandl et al. [48,49] have reported fMRI experiments in which they found increased *FA* value during and after the performance of a task, and proposed that swelling of the glial cells may be the mechanism underlying the observed increase in the value of *FA*. This observation appears to contradict an anticipated decrease in the *FA*-value produced by cation and water movements perpendicular to axonal membranes conducting action potentials.

Also, over the last decade, MR methods have been developed in attempts to image directly neuronal currents induced during action potentials measuring possible perturbations to the MR signal (magnitude and phase) caused by these currents. Presently, the results obtained in humans by these methods, termed neuronal current MRI (ncMRI) (also, Lorentz effect imaging and encephalographic MRI – eMRI), remain controversial [50,51].

As previously mentioned, current technology does not provide a definitive solution to the problem of imaging currents on axons of firing neurons using MRI. The model we proposed herein seems to capture an important mechanistic aspect of the action potential and neural conduction, which has not been considered so far in the existing literature. The impact of BOLD seems to be the major confounding factor in this research and it may well be that the observed increase in *FA* by Mandl and colleagues reflects changes in BOLD signal as pointed out recently by Autio and Roberts [52]. Our calculations predict that there will be a decrease in FA with increased transport of water through Na^+^/K^+^ channels as a function of action potentials. We realize the need for doing more complicated modeling, especially taking into consideration time dependence of the diffusion equations, however we suggest that this should be the subject of further studies. Future studies attempting to perform the dynamic or functional diffusion imaging experiment should make sure to control for the impact of BOLD signal changes and the effect of hypercapnia on *FA* measures. Furthermore, technological advances in MRI are needed to achieve robust and reliable measure of axonal activity during the performance of a functional task. Thus, performing the measurements at high magnetic fields will improve the signal-to-noise ratio and facilitate the detection of small changes with higher sensitivity. Also, state of the art Connectome diffusion imaging technology [53], using two or three times stronger field gradients as compared to the ones routinely used will allow the use of shorter echo times, and hence higher signal-to-noise ratios and lower BOLD contamination. Combined with optimized data acquisition strategies, i.e., number of gradient orientations and averages, this newly emerging technology may be critical in fulfilling the goal of testing this model.

## Figures and Tables

**Fig. 1:**
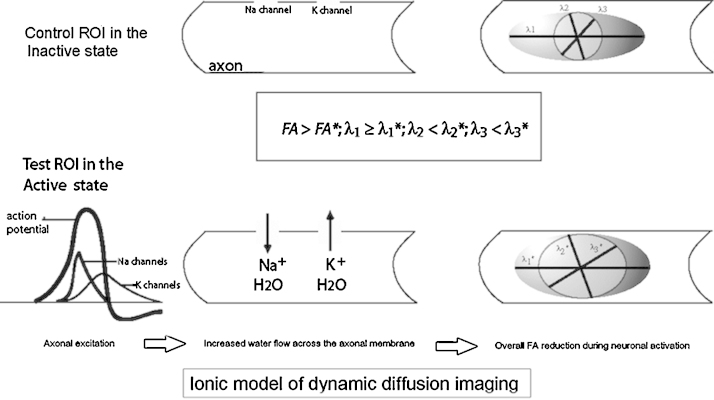
Illustration of the ionic DTI (iDTI) model that reflects the “anisotropy reduction due to axonal excitation” or AREX hypothesis: model of the physiological mechanism underlying axonal and fiber tract excitation and its detectability by DTI. Schematically, it is represented how task-dependent neural stimulation would modify the macromolecular and ionic environments of water molecules of the axonal membrane resulting in increased water flow across the membrane. This anisotropic water flow prevails in the plane perpendicular to the principal axis of the axon and fiber tract and this flow is reflected in a FA reduction and DTI signal attenuation during neuronal activation, such as a functional task. Thus, dynamic DTI (dDTI) provides direct functional measurements of excited axons and excited fiber tracts. Abbreviations: DTI, diffusion tensor imaging; *FA*, fractional anisotropy; *λ*_1_, *λ*_2_, *λ*_3_, eigenvalues of the corresponding diffusion tensor in directions parallel, *λ*_1_, and perpendicular, *λ*_2_ and *λ*_3_, to the main fiber axis; ROI, region of interest.

**Table 1:** Relevant parameters of corticospinal tract model.

Type of axons	Number of axons^a^	Axon diameter^a^ (μm)	Area node ranvier (μm^2^)^b^	Total no. Na^+^ channels (×10^10^)^d^
Myelinated	567,000 (0.550)	2	12.57	125
	105,000 (0.102)	5	31.42	23.1
	28,000 (0.027)	8	50.27	6.2
	30,000 (0.029)	11	69.12	6.6

Unmyelinated	300,000 (0.291)	3	–c	198

a Distribution of axon diameters according to Refs. [12–14]. The total number of axons considered is about 10^6^ and the fraction corresponding to diameters listed in next right column are in parenthesis.

b The length of a node of Ranvier is considered equal to 2 μm [16].

c The mean membrane area per axon for the volume of interest here (voxel of 3.5 mm in length) is 33,000 μm^2^, approximately.

d The density of Na^+^ channels per μm^2^ in a node of Ranvier of a myelinated axon is assumed to be 10,000 [16]. In non-myelinated axons, the surface density of Na^+^ channels per μm^2^ is considered equal to 200 [17].
